# Global Comparative Review of Guidelines for Cervical Adenocarcinoma In Situ

**DOI:** 10.3390/life16030461

**Published:** 2026-03-11

**Authors:** Giovanni Delli Carpini, Camilla Cicoli, Marco Bernardi, Jasmine Saee, Martina Petrini, Valentina Ferrari, Jacopo Di Giuseppe, Luca Giannella, Giuseppe Vizzielli, Andrea Ciavattini

**Affiliations:** 1 Gynecologic Section, Department of Odontostomatologic and Specialized Clinical Science, Università Politecnica delle Marche, 60123 Ancona, Italy; g.dellicarpini@staff.univpm.it (G.D.C.); camilla.cicoli@ospedaliriuniti.marche.it (C.C.); marco.bernardi@ospedaliriuniti.marche.it (M.B.); jasmine.saee@gmail.com (J.S.); petrini.martina@gmail.com (M.P.); ferrari.valentina792@gmail.com (V.F.); jacopo.digiuseppe@ospedaliriuniti.marche.it (J.D.G.); l.giannella@staff.univpm.it (L.G.); 2 Unit of Obstetrics and Gynecology, “Santa Maria della Misericordia” University Hospital, Azienda Sanitaria Universitaria Friuli Centrale, 33100 Udine, Italy; giuseppe.vizzielli@uniud.it

**Keywords:** adenocarcinoma in situ, AIS, guidelines, colposcopy, conservative management, hysterectomy, endocervical curettage, LEEP, cold knife conization, follow-up

## Abstract

Background: The approach to adenocarcinoma in situ (AIS) is challenged by diagnostic complexity, limited high-quality evidence, and heterogeneous guidance. Methods: We conducted a narrative comparative review of global guidelines/recommendations (2012–2025; search updated 1 October 2025), extracting data across 38 topics related to AIS management and classifying indications into five categories of coverage/consensus. Results: Twenty documents from national or supranational bodies were included. A cross-guideline consensus emerged on eight core items (colposcopy for any glandular cytologic abnormality; role of HPV test; mandatory histologic confirmation; excisional treatment for histologic AIS; re-excision when margins are involved; criteria and type of hysterectomy; and expert/centralized management). Operational variability emerged in the excisional technique, pathways for discordant results, management during pregnancy, and follow-up protocols. Divergent guidance was most evident for indications to endocervical sampling, criteria for conservative management, and the need for hysterectomy after completed childbearing. Limited-coverage consensus involved the technique of initial histologic sampling, endometrial assessment, and pathways for cytology subtypes. Several areas remained unaddressed. Conclusions: While the essential management of AIS is well defined, uncertainty increases when treatment must be personalized. A core outcome set and rigorous multicenter studies are needed to reduce heterogeneity and enable truly evidence-based personalization.

## 1. Introduction

Adenocarcinoma in situ (AIS) is a pre-invasive cervical neoplasm arising from the superficial epithelial lining of the endocervical glands, representing the precursor of adenocarcinoma [1,2]. AIS management represents one of the significant modern challenges for gynecologic oncologists and colposcopists due to both epidemiological and clinical aspects. Indeed, while the squamous counterpart appears to be declining over the past few years, mainly due to effective screening methods [3], invasive adenocarcinoma and AIS have shown an increasing trend in incidence (1–2 cases per 100,000 per year) [4,5]. This trend may not be adequately reflected in the available guidelines on cervical cancer prevention. It is also worth noting that approximately 70% of new diagnoses occur before 35 years, with most cases observed between 32 and 40 years [6,7]. All these epidemiological data lead to the need for an early diagnosis, to accommodate the potential childbearing desire of these young patients. One of the primary concerns is the relatively low predictive value of the available first-level tests; indeed, cervical cytology has a roughly estimated sensitivity for glandular lesions as low as 45% [8]. The execution of cervical cytology combined with a human papillomavirus for high-risk types (HR-HPV) test is considered the most accurate method for excluding glandular lesions [9]. However, it is worth noting that up to 15% of AIS may not be associated with HPV infection and may exhibit more aggressive behavior [10]. The importance of first-level test accuracy in the setting of early diagnosis is amplified by the fact that most glandular lesions are asymptomatic and rarely cause symptoms. Glandular lesions tend to grow deep within the endocervical canal and may be covered by normal overlying epithelium without pathognomonic features that can distinguish AIS from other cervical lesions [1,7,11], thus making it more difficult to identify lesions during colposcopic examination [12]. AIS may be incidentally identified in cervical excision specimens obtained for the treatment of squamous intraepithelial lesions [13]. Even at a first AIS diagnosis, a concurrent invasive adenocarcinoma can be identified in up to six percent of cases [14]. Treatment is even more challenging, considering that up to 15% of cases may present “skip-lesions”, characterized by distinct areas of involvement interspersed with at least 2 mm of normal tissue [15]. Although AIS has clinical relevance, its management is primarily guided by a fragmented and limited evidence base, with most studies being retrospective, small-scale, or lacking follow-up at long-term. Given the complexity of AIS management, it is essential to understand how different scientific societies worldwide interpret and incorporate these limitations into their recommendations. To date, a global, AIS-specific, updated comparison of guidelines and recommendations is lacking. The current literature focuses on single nations, invasive cervical cancer, or specific clinical aspects, without addressing guidelines heterogeneity. This review aims to fill this gap by examining the most recent national and international guidelines and recommendations on AIS and offering a comprehensive overview of current global strategies.

## 2. Materials and Methods

A descriptive narrative comparative review of recommendations and guidelines regarding the management of AIS, by a literature search conducted on PubMed/MEDLINE/Scopus on 13 January 2025, focusing on recommendations and guidelines published between 2000 and 2025. The search was re-run on 23 February 2026. The search strategy was the following: “Colposcopy [Mesh]” AND (“Recommendation*”[tiab] OR “Guideline*”[tiab]). Titles and abstracts obtained from the first search were independently screened by two authors (CC and MB) to assess eligibility for full-text review. In cases of disagreement, all selected cases, as determined by at least one reviewer, were retained for full-text evaluation. All guidelines, recommendations, or consensus statements about the diagnosis, management, and follow-up of AIS were included. Narrative reviews and Editorials were excluded, as well as non-institutional documents from the gray literature, which were excluded to ensure comparability and methodological consistency across officially endorsed recommendations. Exclusion criteria also included the availability of more recent versions from the same society and a lack of relevance to AIS diagnosis, management, and follow-up. The same authors also manually searched the websites of two major international societies, the “European Federation for Colposcopy”—EFC and the “International Federation for Cancer Prevention and Colposcopy—IFCPC”, and additionally the National Comprehensive Cancer Network (NCCN) website, to identify their published guidelines or recommendations and retrieve the list of affiliated national scientific societies. Each national society’s website was then consulted individually to identify the most recent documents available worldwide. There were no publishing language limitations. Documents in languages other than English were managed through internal translation. The instrument “AGREE II” was chosen to analyze the quality level of the selected recommendations–guidelines [16].

We selected 38 topics by which the guidelines were compared, grouped in four main topics: first-level tests (management of cytological glandular abnormalities, management by glandular abnormality subtype, role of HPV test, role of HPV genotyping, and role of biomarkers), colposcopy and histological sampling (colposcopic features of glandular lesions, indications for histological sampling, biopsy technique, indications for cervical excision, indications for endocervical sampling, endocervical sampling after cervical excision, endocervical sampling technique, endometrial evaluation/sampling, expert consult, type of cervical excision, surgical techniques for cervical excision, histopathological classification/terminology, pathology reporting standards, and criteria for margin status evaluation), management (discordance between cytology/colposcopy/histology, management after AIS diagnosis, criteria for conservative management of AIS, management after positive margins, adjuvant HPV vaccination, criteria for hysterectomy, type of hysterectomy, surgical technique for hysterectomy, need for lymph node evaluation, hysterectomy after completion of childbearing, special populations (pregnancy), role of imaging, expert review and management, management of occult invasive cancer, and management of HPV-independent AIS), and follow-up (follow-up protocols (test, timing, and duration) after conservative treatment, management of positive results after conservative treatment, follow-up protocols (test, timing, and duration) after hysterectomy, and management of positive results after definitive treatment).

Data from the included guidelines/recommendations were extracted into a predefined comparative table by two authors (GDC and CC). In the case of disagreement, after discussion, another author (AC) made the final decision. The results were qualitatively synthesized by explicitly addressing each topic and highlighting the main similarities and differences between the included guidelines/recommendations. Five qualitative groups were identified to classify the coverage and consensus across the guidelines regarding the 38 identified topics: cross-guideline consensus (indicating that the topic was addressed by all or most guidelines, with substantial agreement regarding the indications), cross-guideline variability (topic addressed by all or most guidelines with minor difference regarding the indications), divergent guidance (topic with significant difference between guidelines in clinical indications), limited-coverage consensus (topic addressed by a limited part of guidelines, with substantial agreement), and unaddressed topics (topics not addressed by any guidelines or from a very small fraction). Since this was a narrative review, no registration was needed.

## 3. Results

**Figure 1:**
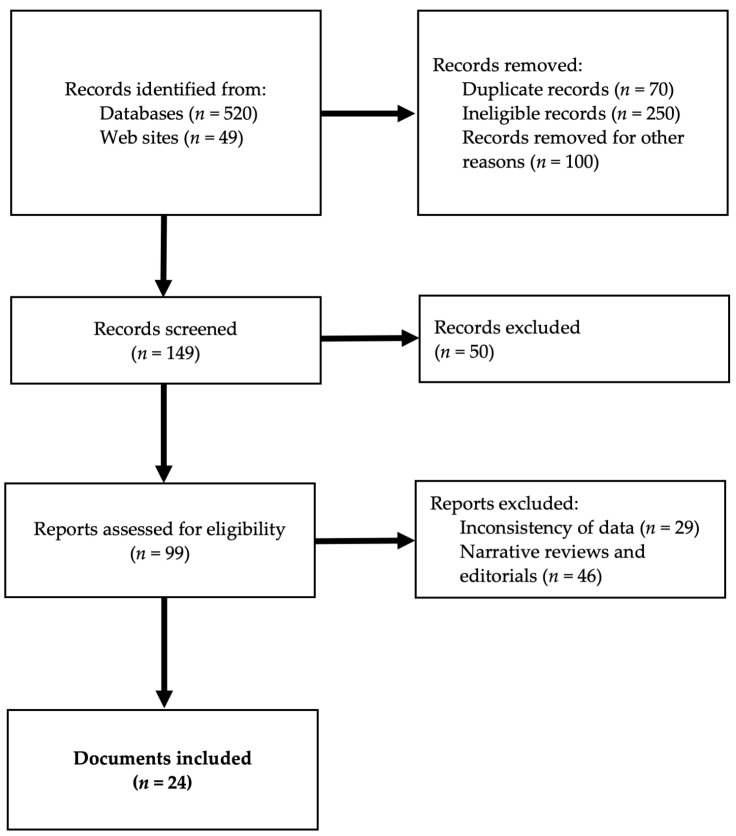
Selection flow of the included studies.

The initial database literature search yielded 569 papers. One hundred twenty-six documents resulted as eligible for full-text review. After the complete assessment, 10 documents were excluded due to the presence of more recent updates. Fifty were excluded because they were duplicates, and 58 items were excluded for lacking relevance to AIS-related topics. The search from the EFC, IFCPC, NCCN, and national societies’ websites yielded 13 additional documents. The selection flow of the included studies is shown in Figure 1.

**Figure 2:**
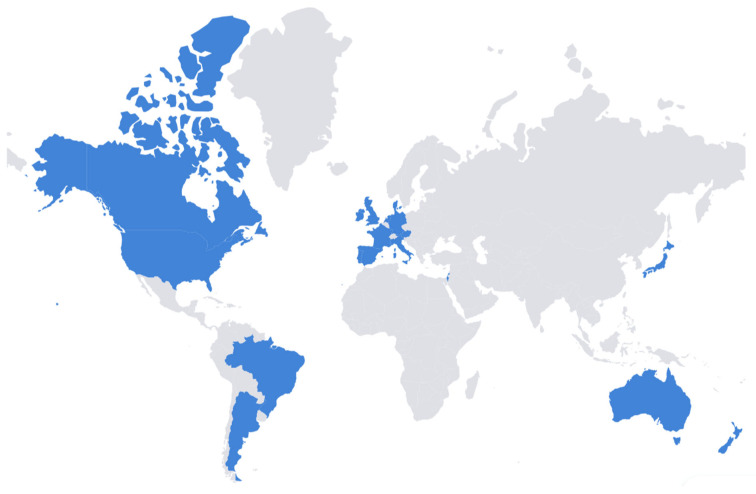
Countries of the included guidelines/recommendation (blue = included, gray = not included).

Twenty-four documents were included in the final evaluation and comparison [7,17,18,19,20,21,22,23,24,25,26,27,28,29,30,31,32,33,34,35,36,37,38,39]. The two sources from France [36,37] and the four from the United States [25,26,38,39] were grouped into a single guideline per country for synthesis. Thus, the final number of included guidelines/recommendations was 20. Figure 2 illustrates the countries represented in the 20 included guidelines/recommendations: four (20%) were from Asia, two (10%) from Australia and New Zealand, 10 (50%) from Europe, two (10%) from North America, and two (10%) from South America.

National or supranational scientific societies issued 14 (70%) guidelines/recommendations, while six (30%) were issued by national health authorities. Supranational guidance (e.g., EFC/ESGO) was considered in the overall comparative framework but was not included in country-based tables (Table 1, Table 2 and Table 3). The year of publishing ranged from 2012 to 2025, with 15 (75%) published in the last five years and 19 (95%) in the last ten years. Supplementary File S1 reports the complete list of countries, the year of publication, and the issuing body for all the included guidelines/recommendations. The results obtained from the AGREE II instrument are synthetized in Supplementary File S2.

### 3.1. First-Level Tests

A cross-guideline consensus emerged on the management of cytological glandular abnormalities and the role of the HPV test. Nineteen (95%) guidelines/recommendations agree that any patient with an abnormal glandular cytological result should undergo colposcopy [7,17,18,19,20,21,22,23,24,25,26,27,28,29,30,35]. The only exception was the French recommendations, stating that HPV-negative patients younger than 30 years with AGC cytology may repeat cervical cytology after three years, while HPV-positive patients should undergo colposcopy [36,37]. HPV testing is not indicated to triage for colposcopic examination in 19 (95%) guidelines/recommendations [7,17,18,19,20,21,22,23,24,25,26,27,28,29,30,31,32,33,34,35,36]. However, its execution is considered beneficial for obtaining additional clinical information in five (25%) guidelines/recommendations [7,21,29,30,31]. A limited-coverage (7/20–35%) consensus emerged regarding management by glandular abnormality subtype, with no substantial difference in the indication for colposcopy across subtypes. The role of biomarkers and the role of HPV genotyping were both unaddressed in all the included guidelines/recommendations, except in the USA recommendations, suggesting an opportunity to perform endocervical sampling in cases of HPV-18 positivity, regardless of colposcopy findings [25,26].

### 3.2. Colposcopy and Histological Sampling

A cross-guideline consensus emerged on the indications for histological sampling, which is deemed necessary from all the included guidelines/recommendations [7,17,18,19,20,21,22,23,24,25,26,27,28,29,30,31,32,33,34,35,36], and on the need for histological confirmation following positive or suspicious cytology for glandular pathology through cervical excision, reported from 15 (75%) guidelines/recommendations [7,17,21,23,24,25,26,27,29,30,32,33,34,35,36,37].

A limited-coverage consensus was noted about three topics: the need to perform an endocervical sampling following diagnostic excision (5/20–25%) [7,17,21,24,25,26], the indication to incorporate hysteroscopic evaluation with endometrial biopsy as part of the diagnostic pathway (10/20–50%), particularly in patients over 35 years presenting with symptoms suggestive of endometrial pathology or with specific risk factors (e.g., obesity and polycystic ovarian syndrome) [7,17,19,20,21,24,27,29,31,35], and the importance that experienced colposcopists should perform colposcopy, ideally within specialized centers, with second opinions encouraged when diagnostic uncertainty persists (5/20–25%) [7,17,24,29,31].

Colposcopic features of glandular lesions were an unaddressed topic, with the exception of EFC/ESGO, indicating that most lesions are localized in the canal or in proximity to the squamo-columnar junction (SCJ), with non-specific features that may be potentially confused with immature metaplasia [24]. Additional reported colposcopic signs of AIS or invasion are nodularity and coalescence of glandular papillae [24]. The endocervical sampling technique was described only in the USA-ASCCP guidelines [38]. No guidelines/recommendations provide specific criteria on which histopathological classification/terminology, pathology reporting standards, or histological criteria for margin status evaluation should be used.

Divergent guidance emerged about the biopsy technique, with five (25%) guidelines/recommendations allowing punch biopsy [22,27,32,35,36,37], five (25%) indicating its role only in the case of suspicion of invasive disease, and one (5%) only when a diagnostic excision is not feasible [33]. Three (15%) guidelines/recommendations do not recommend targeted punch biopsy [17,24,28]. The indications for endocervical sampling were also divergent: it is considered a key diagnostic step in 13 (65%) guidelines/recommendations [7,17,18,19,20,21,22,24,25,26,30,31,33,34,35,36,37], but its routine use is not supported by three (15%) [18,27,28], and it is not recommended by the United Kingdom (UK) guidelines [28].

Cross-guideline variability emerged regarding the type of cervical excision. A general agreement was noted on the need to individually assess the extent of excision, with a preference for a type 3/cylindrical excision tailored to the visibility of the SCJ and the fertility desire. The reported optimal cervical length ranged from 10 to 25 mm, with a free margin of 3–10 mm. Table 1 details the specific indications for each included guideline/recommendation. The surgical techniques for cervical excision also presented cross-guideline variability, with recommendations to prefer the loop electrosurgical excision procedure (LEEP/LLETZ) or cold-knife conization (CKC), according to the experience or preference of the operator, with only two (10%) guidelines/recommendations not considering LEEP/LLETZ as adequate due to thermal damage [22,23]. On the other hand, the French recommendations specify LEEP/LLETZ as the sole technique for cervical excision [36,37]. Laser conization was permitted by three (15%) guidelines/recommendations [7,19,20], and one (5%) also mentions radiofrequency needle excision [7]. No guidelines recommend the use of ablative techniques [7,17,18,19,20,21,22,23,24,25,26,27,28,29,30,31,32,33,34,35,36]. Table 1 reports the indication for each included guideline/recommendation. All guidelines/recommendations report the need to obtain a single intact specimen with clearly evaluable margins [7,17,18,19,20,21,22,23,24,25,26,27,28,29,30,31,32,33,34,35,36].

### 3.3. Management

Cross-guideline consensus emerged about management after AIS diagnosis (cervical excision procedure as the first-line approach) [7,17,18,19,20,21,22,23,24,25,26,27,28,29,30,31,32,33,34,35,36], management of positive margins after cervical excision (second excision to achieve negative margins and exclude invasive adenocarcinoma [7,17,18,19,20,21,22,23,24,25,26,27,28,29,30,31,32,33,34,35,36]; total hysterectomy when the margins are positive after a second excision, in case of refusal of a second excision by the patient, or if a second excision cannot be performed [7,17,18,19,20,21,22,23,24,25,26,27,29,30,31,32,33,34,35,36]; and the UK guidelines are the only ones that provide for follow-up even in cases of positive margins [28]), criteria for hysterectomy (treatment of choice for patients who have completed their reproductive plans [7,17,18,19,20,21,22,23,24,25,26,27,28,30,31,32,33,34,35,36]; Australia is the only country that explicitly discourages routine hysterectomy, favoring an individualized approach [29]), type of hysterectomy (Table 2), and expert review and management (treatment centralization in high-volume or specialized centers [7,17,19,20,21,24,25,26,31]).

Adjuvant HPV vaccination, surgical technique for hysterectomy (laparoscopic, vaginal, laparotomic, or robotic), need for lymph node evaluation, role of imaging, and management of HPV-independent AIS were unaddressed among all included guidelines/recommendations.

Cross-guideline variability emerged about the management of discordance between cytology, colposcopy, and/or histology, a topic addressed by 13 (65%) guidelines/recommendations [7,17,18,19,20,25,26,27,28,29,30,31,35,36,37]. The specific recommendations are reported in Table 2.

Divergent guidance was noted about the conservative management of AIS. Fifteen (75%) guidelines/recommendations indicate that a conservative management may be chosen for patients desiring fertility if, after excisional treatment, the margins and endocervical sampling are negative, invasive adenocarcinoma is excluded, and they agree to undergo strict follow-up [7,17,18,19,20,21,24,27,29,30,31,32,35,36,37], while two (10%) advise against conservative treatment even in patients desiring fertility, because of the high risk of recurrence, including invasive adenocarcinoma [22,23]. Hysterectomy is reported as mandatory at the end of reproductive desire in nine (45%) guidelines/recommendations [19,20,24,25,26,27,33,34,35,36,37] and preferable in seven (35%) [7,17,21,28,30,31,32]. According to the Australian guidelines, indications for hysterectomy include persistently positive margins after multiple excisions or impossibility of further excision, difficulties in ongoing surveillance (absent or stenotic cervical os), and patient anxiety [29]. Additional indications for hysterectomy are also reported, even in patients wishing to preserve fertility. These include persistently positive margins or positive endocervical sampling after multiple excisions or impossibility of further excision [7,17,18,19,20,21,22,23,24,25,26,27,28,29,30,31,32,33,34,35,36], and difficulties in colposcopic follow-up due to cervical stenosis, patient anxiety, or poor compliance [7,17,24,29,32].

Fourteen (70%) guidelines address the management of glandular lesions during pregnancy [7,17,18,19,20,21,23,24,25,26,27,29,31,34,36]. Substantial consensus emerged about the direct referral to colposcopy to rule out invasive disease, prohibition of endocervical or endometrial sampling during pregnancy (with the exception of Portuguese guidelines [21]), biopsy execution only in cases of suspected invasive carcinoma [7,17,18,19,20,21,22,23,24,25,26,27,28,29,30,31,32,33,34,35,36], cytological and colposcopic follow-up during pregnancy (every 12 weeks according to Portuguese and Spanish guidelines [21,31]), and treatment of lesions without suspicion of invasion after delivery. The timing of treatment ranged from 8–12 weeks to 3–6 months after delivery [17,24,27,36]. A diagnostic excision was permitted by seven (35%) guidelines/recommendations for the suspicion of invasive adenocarcinoma [17,18,19,20,23,24,25,26]. Austrian and German recommendations advise performing diagnostic excision between the 16th and the 20th week of gestation by loop excision combined with bipolar coagulation, without the need for prophylactic cerclage, unless the cervix is already significantly shortened [19,20]. Israeli guidelines recommend performing the excision before the 15th week of gestation [34]. A limited-coverage consensus (9/20–45%) emerged about the management of occult invasive adenocarcinoma, with the indication for immediate referral to a gynecologic oncology center [7,19,20,21,22,27,29,30]. The American guidelines (SGO) recommend, in cases of microinvasive adenocarcinoma (stage IA1–IB1), surgical management by means of simple hysterectomy and pelvic lymphadenectomy [26].

### 3.4. Follow-Up

All topics related to follow-up presented cross-guideline variability. Table 3 reports the specific indication for test, timing, duration, and management of positive results, both after conservative and definitive treatment.

**Figure 3:**
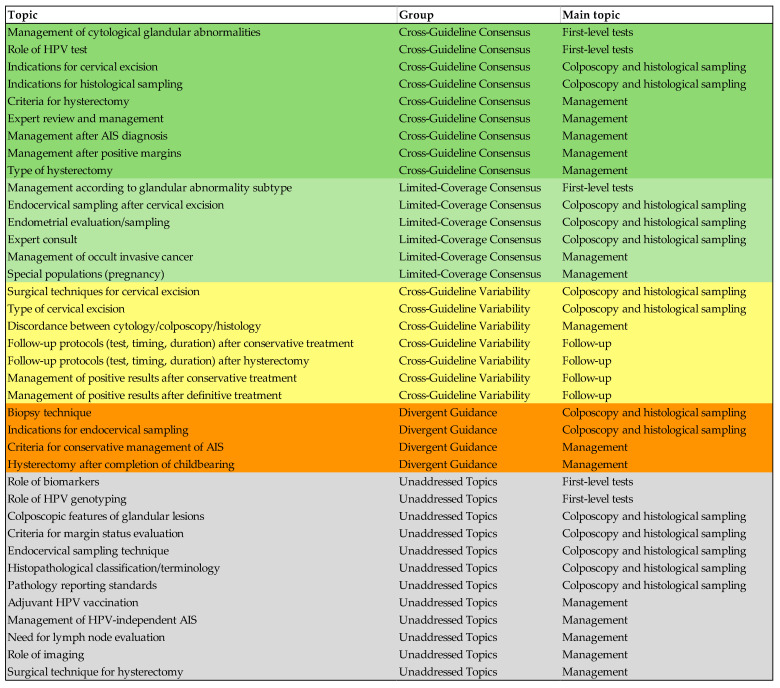
Overview of the 38 AIS-related topics across guidelines/recommendations, categorized by: cross-guideline consensus (dark green), limited-coverage consensus (light green), cross-guideline variability (yellow), divergent guidance (orange), and unaddressed topics (gray).

Table 4 provides a comparative overview of the most clinically relevant AIS recommendations from oncologic and supranational societies, focusing on key management decisions. Figure 3 illustrates the attribution of each of the 38 identified topics to the respective qualitative groups of coverage and consensus across the guidelines.

## 4. Discussion

The comparison of global guidelines/recommendations on AIS enabled us to identify the cornerstones of management, areas of variability, and divergent topics, thereby highlighting the practical implications, identifying key areas for future research, and providing insights into this area of healthcare organization.

A cross-guideline consensus on eight core items (management of cytological glandular abnormalities, role of HPV test, indications for histological sampling, management after AIS diagnosis, management of positive margins, criteria for hysterectomy, type of hysterectomy, and expert review and management) was noted. The consensus on these topics reflects standardized management, based on expert opinion and evidence from the literature, as synthesized in available systematic reviews [15,40,41,42].

Several analyzed topics showed cross-guideline variability, limited consensus, or divergent guidance between guidelines/recommendations. The sources of heterogeneity can be grouped into: (i) clinical setting and healthcare system organization; (ii) operator-related factors and clinical expertise; (iii) patient-related factors and decision-making context; and (iv) evidence-based and methodological factors.

The clinical setting and healthcare system organization group includes resource availability, access to care, centralization, and high-quality colposcopy, the screening modality (HPV test vs. cytology), and the HPV vaccination/screening coverage. These factors may explain why some recommendations may be feasible in some countries but not in others, and account for differences even when formal guidelines/recommendations are similar. Items that may be affected by these factors are the management of discordant results (cross-guideline variability) and follow-up protocols. Countries with HPV-based screening usually consider the HPV test as a more reliable predictor of recurrence risk, and follow-up protocols that already include the HR-HPV test may be more easily implemented for glandular lesions. Differences in HPV vaccination/screening coverage may also add further complexity: countries with high coverage seem to favor personalized strategies, whereas those with limited coverage may adopt more standardized approaches [43,44]. The lack of clear follow-up protocols is particularly clinically relevant, as a recent meta-analysis found that AIS recurrences are more frequent after conservative treatment than after hysterectomy, with a risk ratio of 8.44 (95% CI 3.36–21.19) and a risk of invasive cancer estimated between 1 and 2% [3].

The operator-related factors and clinical expertise comprise operator training and experience, local expert opinions, and local practice. Variability in training standards and operator experience may lead to differences in diagnostic accuracy and treatment adequacy, affecting national guidelines and recommendations [43,44]. More specifically, these factors may determine the differences in cervical excision surgical technique, management during pregnancy, and the indications for endocervical sampling. Indeed, both CKC and LEEP are reported as alternative techniques for cervical excision, with different emphases on their advantages and disadvantages. Since Jiang et al. demonstrated that there is no difference in safety and effectiveness between LEEP/LLETZ and CKC for the conservative management of AIS [42], the observed difference may be related to the uneven availability of adequately trained operators in difficult surgical procedures. Expert operators should perform colposcopy during pregnancy to recognize pregnancy-related changes and implement timely, appropriate management, and this level of training is not uniform across countries [24]. Regarding endocervical sampling, since AIS-specific evidence on diagnostic accuracy is lacking, the range of recommendations, from routine to selective use, is likely due to local practice and expert opinion. Moreover, performing endocervical sampling and interpreting its results requires skills that are not uniformly distributed across healthcare systems [45].

Patient-related factors and decision-making context may encompass both patient values (e.g., reproductive preferences or age at first pregnancy) and medico-legal pressures. The influence of these factors is particularly evident on the criteria for conservative management of AIS, and on the need to perform hysterectomy once childbearing is complete. For conservative management, no difference in the risk of invasive recurrence compared with hysterectomy was reported [3]. However, the need for accurate follow-up due to the higher incidence of AIS recurrence is emphasized [3]. In this context, medico-legal pressures may influence recommendations in countries with high litigation risk. Considering the lower thresholds for acceptable uncertainty, clinicians may have a stronger inclination to recommend hysterectomy once childbearing is complete. The age at first pregnancy may also play a significant role: the trend of delayed childbearing observed in numerous countries may influence a stronger recommendation for a conservative approach. The need for hysterectomy after completion of childbearing lacks robust data to be defined as a standard management of patients with an AIS diagnosis. Therefore, some guidelines may be driven by a more precautionary interpretation of recurrence risk, while others support a personalized approach based on AIS-specific characteristics (e.g., multifocality), margin status, adherence to follow-up, and patient preferences, further illustrating how cultural and medico-legal environments may shape clinical decision-making.

The methodological heterogeneity across guidelines was highlighted by the application of the AGREE II instrument (Supplementary File S2), particularly in two domains: Rigor of development and Applicability. This heterogeneity partially explains the differences observed in clinical indications and implies careful consideration in treating them as equivalent in terms of methodological soundness.

Topics with limited-coverage consensus included the technique of the first histological sampling, the need for endometrial evaluation or sampling, and management according to the subtype of glandular abnormality. These areas would benefit from standardizing terminology, decision thresholds, and adequacy criteria to reduce unwarranted variability.

Several unaddressed topics emerged, including the usefulness of HPV genotyping and biomarkers, the significance of HPV-independent AIS, the colposcopic features of glandular lesions, and the endocervical sampling technique. Regarding the role of HPV genotyping, evidence is needed to evaluate its potential for stratifying patient risk of AIS, given that there is no specific guidance on the best method in this context. HPV-independent AIS was unaddressed across guidelines/recommendations; given its distinct biology, potentially more aggressive behavior, and limited performance of HPV-based strategies, explicit guidance and dedicated pathways are needed [46]. Some of these omissions are likely implicit non-indications (e.g., imaging or nodal evaluation), but making these indications explicit would improve completeness and transferability, as well as help prioritize future research. To date, the existing literature on these topics is scarce and does not allow for specific recommendations. Strengthening this research area is crucial to permit the definition of additional recommendations in the future.

Overall, national guidelines appear largely aligned with the core management principles outlined by oncologic and supranational societies (Table 4), although differences emerge in areas requiring individualized decision-making, particularly regarding conservative management and follow-up intensity.

Regional differences in cervical adenocarcinoma incidence have been reported, particularly in parts of East and Southeast Asia [4]. Although geographically stratified data specific to AIS remain limited, some national guidelines from this region (e.g., Hong Kong and Singapore) appear to adopt a relatively more cautious or radical approach, including a preference for cold-knife conization and stricter indications for conservative management. While no causal inference can be drawn, these observations raise the possibility that regional epidemiological patterns and perceived recurrence risk may subtly influence therapeutic thresholds.

The future strategy to reduce heterogeneity across countries needs a standard definition of core outcomes, including margin status, re-treatment, criteria for a conservative approach, recurrence, adherence to follow-up, adverse events, and patient-reported outcomes. Secondly, well-conducted, multicenter, and rigorous clinical studies are needed to ground future recommendations in robust evidence. In addition, establishing an international consortium dedicated to AIS—capable of coordinating multicenter prospective studies, standardizing training, promoting shared registries, and updating recommendations—would be a crucial step toward achieving cohesive, evidence-based global management.

This study presents some limitations. Firstly, the categorization of consensus levels entails a degree of subjective judgment. Secondly, our search was limited to guidelines/recommendations accessible through bibliographic databases and scientific society websites, potentially underrepresenting low- and middle-income countries. Third, local or institutional protocols and non-institutional recommendations were not included, despite their potential influence on everyday clinical practice. Additional limitations relate to the heterogeneity of guideline scope, structure, and update frequency. Finally, since the authors translated guidelines published in languages other than English internally, a limited risk of interpretative bias may have been introduced.

## 5. Conclusions

The essential management of AIS is well defined for a straightforward pathway, with consistency in recommendations regarding core items. These items may be considered the international minimum threshold in AIS management. Uncertainty increases when treatment must be personalized, in the case of young patients with fertility desire, discordance between cytology, colposcopy, and histology, or when the most appropriate follow-up strategy should be defined. In these scenarios, decisions and indications often rely on local expertise and customary practice, considering the limited available evidence. This variability may affect clinical outcomes and patient compliance. To reduce heterogeneity, an international consensus is needed to define core outcomes and develop recommendations. Moreover, the conduct of targeted, rigorous, multicenter studies of a few critical aspects (standardization of follow-up after conservative management, shared clinical outcomes, and HPV-independent AIS) should be a future priority for the research and clinical community, enabling truly evidence-based personalization. Clinicians, researchers, and policymakers should collaborate to implement this roadmap to improve outcomes for patients affected by AIS.

## Figures and Tables

**Table 1:** Colposcopy and sampling.

Country	Type of Cervical Excision	Surgical Techniques for Cervical Excision
Argentina [35]	Technical criteria not specified	Surgical technique not specified
Australia [29]	Type 3 excision (>15 mm of endocervical tissue); type 2 if age under 35 years (up to 15 mm of endocervical tissue)	Cold-knife as gold standard; LEEP by highly experienced operators
Austria [19]	Resection with narrow tumor-free margins; larger cones if no reproductive desire	Laser conization allowed
Brazil [27]	Technical criteria not specified	Surgical technique not specified
Canada [17]	Type 3 excision of entire TZ, at least 15 mm in length, deeper excisions for peri- or postmenopausal patients	Top-hat unacceptable
Denmark [32]	Wide excision	Surgical technique not specified
France [36,37]	Not specified technical criteria	LEEP/LLETZ
Germany [20]	Resection with narrow tumor-free margins; larger cones if no reproductive desire	Laser conization allowed
Hong Kong, China [23]	Length of at least 10 mm where feasible	Cold-knife conization; LEPP/LLETZ inadequate; top-hat unacceptable
Israel [34]	Technical criteria not specified	Surgical technique not specified
Italy [7]	Type 3 excision, entire TZ, 10–15 mm of endocervical canal if adequate colposcopy or 20–25 mm of endocervical canal if not visible SCJ or age ≥ 50 years	Operator’s choice according to most confident technique (CKC, laser, LEEP, radiofrequency needle); top-hat unacceptable
Japan [33]	Not specified technical criteria	Not specified technical criteria
The Netherlands [18]	Not specified technical criteria	Not specified technical criteria
New Zealand [30]	Not specified technical criteria	Not specified technical criteria
Portugal [21]	Not specified technical criteria	Not specified technical criteria
Singapore [22]	Not specified technical criteria	Cold-knife conization; LEPP/LLETZ inadequate; top-hat unacceptable
Spain [31]	Not specified technical criteria	Not specified technical criteria
United Kingdom [28]	Cylindrical excision, entire TZ, at least 10 mm length above SCJ (young patients/wish to preserve fertility/visible SCJ), or 20–25 mm of the endocervical canal (older patients/not visible SCJ)	Not specified technical criteria
USA [25,26,38,39]	Specimen length at least 10 mm or 18–20 mm in patients without desire for future pregnancies	Top-hat unacceptable

**Table 2:** Management.

Country	Discordance Between Cytology/Colposcopy/Histology
Argentina [35]	Annual co-testing plus colposcopy for two years, or cytology plus colposcopy (every six months for two years). If negative, return to national screening. In cases of strong clinical suspicion, diagnostic excision.
Australia [29]	Referral to colposcopy. If not-visible lesion or type 1/2 transformation zone, transvaginal ultrasound and endometrial biopsy over 45 years or over 35 years with risk factors. If negative, co-testing at six and 12 months. In case of type 2/3 transformation zone, consider diagnostic excision over 45 years with completed childbearing or particular concern about the risk of cancer.
Austria [19]	Co-testing, colposcopy with endocervical curettage, and transvaginal ultrasound within a three-month period. If negative, hysteroscopic assessment and possibly a diagnostic cervical excision (postmenopause) or follow-up with co-testing, colposcopy, and repeat endocervical sampling after six months and then one year or diagnostic conization in case of strong suspicion (premenopause). Diagnostic excision in case of positive HPV test with negative histology for postmenopausal patients
Brazil [27]	Hysteroscopic assessment with endometrial biopsy. If negative, follow-up with cervical cytology every six months for one year, then cytology every three years.
Canada [17]	Annual co-testing for two years. If negative, HPV-based screening every five years.
Denmark [32]	Not addressed
France [36,37]	If glandular disease at cytology and negative HPV, repeat cytology (under 30 years) or HPV testing (over 30 years) after three years. If negative, co-testing at one year. If negative, HPV screening every three years.
Germany [20]	Co-testing, colposcopy with endocervical curettage, and transvaginal ultrasound within a three-month period. If negative, hysteroscopic assessment and possibly a diagnostic cervical excision (postmenopause) or follow-up with co-testing, colposcopy, and repeat endocervical sampling after six months and then one year or diagnostic conization in case of strong suspicion (premenopause). Diagnostic excision in case of positive HPV test with negative histology for postmenopausal patients
Hong Kong, China [23]	Not addressed
Israel [34]	Not addressed
Italy [7]	Cytological slide review. If glandular abnormality confirmed, colposcopy with diagnostic excision.
Japan [33]	Not addressed
The Netherlands [18]	Repeat HPV test + cervical cytology at three to six months. Diagnostic excision in case of strong clinical suspicion.
New Zealand [30]	Cytological slide review. If negative, management in a multidisciplinary center.
Portugal [21]	Not addressed
Singapore [22]	Not addressed
Spain [31]	In case of AGC-NOS, co-testing at one year twice, with return to national screening if consistently negative. For AGC-H, diagnostic cervical excision.
United Kingdom [28]	Referral to a multidisciplinary center. Co-testing at six months, with return to screening if negative.
USA [25,26,38,39]	Co-testing at one year twice, with return to co-testing every three years if negative.

**Table 3:** Follow-up.

Country	Test, Timing, and Duration After Conservative Management	Management of Positive Results After Conservative Management	Test, Timing, Duration, Management of Positive Results After Definitive Management
Argentina [35]	Co-testing + ECC. Every six months for 3 years, then annually for 2 years. Then, three-yearly co-testing for at least 25 years	No information provided	Not specified
Australia [29]	Co-testing. Annually for five years, then every three years for at least 25 years	No information provided	Same as conservative treatment
Austria [19]	Co-testing. Timing and duration not defined—strict surveillance	No information provided	Not specified
Brazil [27]	Cytology after six and 12 months, then each year for five years. Subsequent return to routine screening indefinitely	Second conization or total hysterectomy if positive cytology	Cytology for five years, then every three years indefinitely
Canada [17]	HPV test, with colposcopy and ECC after six and 18 months, then each year for three years. Then, return to routine screening indefinitely	No information provided	Not specified
Denmark [32]	Co-testing each six months for two years, then each year for three years. Subsequent return to routine screening	HPV positivity: surveillance with co-testing each six months for two years. Diagnostic excision or total hysterectomy if positive co-testing	Not specified
France [36,37]	HPV test within six months, then annually. ECC if positive HPV. Duration not specified	Colposcopy if HPV positivity. If negative, annual HPV testing. If unsatisfactory, diagnostic excision. If positive, management according to histopathological result.	HPV testing at six months: if negative, return to three-yearly HPV-based screening indefinitely. If positive, referral to colposcopy.
Germany [20]	Co-testing. Timing and duration not defined—strict surveillance	No information provided	No information provided
Hong Kong, China [23]	Not clearly defined	No information provided	No information provided
Israel [34]	Not clearly defined	No information provided	No information provided
Italy [7]	Co-testing + ECC each six months for two years, then each year for three years. Then, annual cytology indefinitely	Diagnostic conization before hysterectomy if AIS recurrence	Same as conservative treatment
Japan [33]	Not clearly defined	No information provided	No information provided
The Netherlands [18]	Co-testing at six months, then annual for at least five years	Diagnostic excision if HPV positivity	Same as conservative treatment
New Zealand [30]	Annual co-testing for five years, then every three years for at least 25 years	No information provided	Not specified
Portugal [21]	Co-testing + ECC every six months for three years, then annual for two years. Then three-yearly co-testing for at least 25 years	Referral to colposcopy with ECC if positive cytology/HPV test. Conization and/or total hysterectomy if AIS diagnosis	Co-testing at six and 12 months after definitive treatment, then annually indefinitely. If positive, vaginal colposcopy is indicated. If negative cytology is negative with positive HPV, repeat co-testing at six and 12 months
Singapore [22]	Test and timing not specified. Continuation of screening for at least twenty years	No information provided	No information provided
Spain [31]	Co-testing every six months for three years, then semi-annual cytology, colposcopy, and ECC test for three years, then annual HPV test for two years. Subsequent annual co-testing for at least 25 years	Diagnostic conization before hysterectomy if AIS recurrence	Routine screening for at least 25 years after definitive treatment
United Kingdom [28]	Co-testing at six, 12, and 18 months, then three-yearly screening	Referral to colposcopy and diagnostic excision according to clinical discretion. If it is not indicated, TOC for a period of ten years.	Not specified
USA [25,26,38,39]	Co-testing + ECC every six months for three years, then annual for two years. Then, three-yearly co-testing indefinitely	Hysterectomy after completed childbearing if HPV positive.	Not specified

**Table 4:** Comparative overview of AIS recommendations from oncologic and supranational societies.

Topic	EFC/ESGO [24]	SGO [26]	NCCN [39]	ASCCP [25]
Cervical excision	Required	Required	Required	Required
Technique of cervical excision	Type 3 excision; at least 10 mm length (fertility desire/visible SCJ), or 18–20 mm (no fertility desire/not visible SCJ). No top-hat; no ablative technique	Specimen length at least 10 mm or 18–20 mm in patients without desire for future pregnancies. Top-hat unacceptable	Specimen length at least 10 mm or 18–20 mm in patients without desire for future pregnancies	Specimen length at least 10 mm or 18–20 mm in patients without desire for future pregnancies
Endocervical sampling	Key diagnostic step	Key diagnostic step	-	Key diagnostic step
Conservative management	Negative margins and endocervical sampling; invasion excluded; agree to strict follow-up	Negative margins; agree to strict follow-up	-	Negative margins and endocervical sampling; agree to strict follow-up
Hysterectomy	Preferred if no fertility desire	Preferred if no fertility desire	-	Preferred if no fertility desire
Follow-up	Co-testing at six (+ECC) and 18 months, then three-yearly screening, with the addition of colposcopy	Co-testing + ECC every six months for three years, then annual for two years. Then, three-yearly co-testing indefinitely (at least 25 years)	-	Co-testing + ECC every six months for three years, then annual for two years. Then, three-yearly co-testing indefinitely

## Data Availability

No new data were created for the present paper.

## Supplementary Materials

The following supporting information can be downloaded at: https://www.mdpi.com/article/10.3390/life16030461/s1, Supplementary File S1—Country, year of publication, and issuing body of the included guidelines/recommendations; Supplementary File S2—AGREE II results for each included guideline/recommendation.

## Abbreviations

The following abbreviations are used in this manuscript:

AIS	Adenocarcinoma in situ
AGC	Glandular cell abnormalities
AGC-FN	Glandular cell abnormalities—favor neoplastic
AGC-H	Glandular cell abnormalities—high grade
AGC-NOS	Glandular cell abnormalities—not otherwise specified
BMI	Body mass index
CKC	Cold-knife conization
EFC	European Federation for Colposcopy
HR-HPV	High-risk human papillomavirus
IFCPC	International Federation for Cancer Prevention and Colposcopy
LEEP	Loop electrosurgical excision procedure
LLETZ	Large loop excision of the transformation zone
PCOS	Polycystic ovarian syndrome
SCJ	Squamocolumnar junction
TOC	Test-of-cure
TZ	Transformation zone
UK	United Kingdom
USA	United States of America
