# Supplementary material for: Global Comparative Review of Guidelines for Cervical Adenocarcinoma In Situ
Source: Life (Basel). 2026 Mar 11;16(3):461. doi: 10.3390/life16030461 (PMC13028419; doi:10.3390/life16030461)
Supplement: Supplementary file 1 [file life-16-00461-s001.zip › Supplementary File 1.pdf]

Supplementary File 1 – Country, year of publication, and issuing body of the included guidelines/recommendations

Country	Year	Issuing body
Argentina [35]	2022	"Sociedad Argentina De Patología Del Tracto Genital Inferior Y Colposcopía – SAPTGIyC", "Sociedad de Obstetricia y Ginecología de Bs. As. (SOGIBA)", "Federación Argentina de Sociedades de Ginecología y Obstetricia – FASGO"
Australia [29]	2025	"National Cervical Screening Program Guidelines - Cancer Council Australia"
Austria [19]	2020	"Österreichische Gesellschaft für Gynäkologie und Geburtshilfe – OEGGG", "Austrian Working Group for Gynecological Oncology – AGO", "Colposcopy Working Group – AGK", "Austrian Society of Cytology – ÖGZ"
Brazil [27]	2016	"Ministério da Saúde - Instituto Nacional de Câncer José Alencar Gomes da Silva – INCA"
Canada [17]	2023	"Gynecologic Oncology Society of Canada – GOC", "Society of Colposcopists of Canada (SCC)"; "Canadian Partnership Against Cancer – CPAC"
Denmark [32]	2021	"Danish Health Authority"
EFC/ESGO [24]	2023	"European Federation for Colposcopy - EFC; European Society of Gynaecological Oncology – ESGO"
France [36,37]	2019-2024	"Institut National du Cancer (INCA)", "Société Française de Colposcopie et de Pathologie Cervico-Vaginale – SFCPCV"
Germany [20]	2020	"Arbeitsgemeinschaft Zervixpathologie und Kolposkopie – AGCPC", "The German Society of Gynecology and Obstetrics – DGGG", "Arbeitsgemeinschaft Prävention und integrative Onkologie - PRIO, DKG Sektion B, Arbeitsgemeinschaft für gynäkologische Onkologie der DKG – AGO"
Hong Kong, China [23]	2024	"The Hong Kong College of Obstetricians and Gynaecologists – HKCOG"
Israel [34]	2022	"Israel Society of Colposcopy and Cervical Pathology – ISCPC"
Italy [7]	2019	"Società Italiana di Colposcopia e Patologia Cervico Vaginale – SICPCV"
Japan [33]	2017	"Japan Society of Gynecologic Oncology – JSGO"
Netherlands [18]	2012	"Federatie Medisch Specialisten"
New Zealand [30]	2020	"National Cancer Screening Program - NCSP; New Zealand Guidelines Group – NZGG"

Portugal [21]	2023	“Secção Portuguesa de Colposcopia e Patologia do Trato Genital Inferior - Sociedade Portuguesa de Ginecologia - SPCPTGI – SPG”
Singapore [22]	2025	“Society for Colposcopy and Cervical Pathology of Singapore – SCCPS”
Spain [31]	2025	“Asociación Española de Patología Cervical y Colposcopia – AEPCC”
United Kingdom [22]	2025	“National Health Service: NHS England, NHS Scotland, NHS Wales, Health and Social Care in Northern Ireland”
USA [25,25,38]	2019<br>2020<br>2023	“American Society of Colposcopy and Cervical Pathology – ASCCP”<br>“Society of Gynecologic Oncology – SGO”<br>“National Cancer Institute/ASCCP Consensus Stakeholders Group”
