# Supplementary material for: Global Comparative Review of Guidelines for Cervical Adenocarcinoma In Situ
Source: Life (Basel). 2026 Mar 11;16(3):461. doi: 10.3390/life16030461 (PMC13028419; doi:10.3390/life16030461)
Supplement: Supplementary file 1 [file life-16-00461-s001.zip › Supplementary File 2.pdf]

Supplementary File 2 – AGREE II results for each included guideline/recommendation

Country	Scope and purpose	Stakeholder involvement	Rigor of development	Clarity of presentation	Applicability	Editorial independence
Argentina [35]	100%	67%	42%	100%	67%	100%
Australia [29]	100%	89%	100%	94%	92%	100%
Austria [19]	100%	78%	83%	78%	67%	100%
Brazil [27]	100%	78%	79%	78%	67%	100%
Canada [17]	100%	89%	96%	100%	87%	100%
Denmark [32]	100%	72%	64%	55%	45%	100%
EFC/ESGO [24]	100%	78%	97%	94%	83%	100%
France [36,37]	100%	78%	52%	67%	58%	100%
Germany [20]	100%	78%	83%	78%	67%	100%
Hong Kong, China [23]	100%	72%	54%	72%	67%	100%
Israel [34]	100%	61%	16%	55%	12%	83%
Italy [7]	100%	89%	94%	100%	92%	100%
Japan [33]	100%	100%	95%	77%	70%	100%
Netherlands [18]	100%	66%	58%	77%	45%	58%
New Zealand [30]	100%	88%	81%	100%	80%	100%
Portugal [21]	100%	72%	64%	77%	54%	100%
Singapore [22]	100%	50%	31%	83%	45%	75%
Spain [31]	100%	100%	89%	94%	87%	100%
United Kingdom [22]	100%	67%	37%	78%	42%	50%
USA [25,25,38]	100%	89%	96%	94%	79%	100%
